# *Ginkgo biloba* L. Prevents Hypobaric Hypoxia–Induced Spatial Memory Deficit Through Small Conductance Calcium-Activated Potassium Channel Inhibition: The Role of ERK/CaMKII/CREB Signaling

**DOI:** 10.3389/fphar.2021.669701

**Published:** 2021-07-12

**Authors:** Neetu Kushwah, Vishal Jain, Manisha Kadam, Rahul Kumar, Aastha Dheer, Dipti Prasad, Bhuvnesh Kumar, Nilofar Khan

**Affiliations:** ^1^ Department of Neurobiology, Defence Institute of Physiology and Allied Sciences (DIPAS), DRDO, Delhi, India; ^2^ Department of Neurophysiology, Defence Institute of Physiology and Allied Sciences (DIPAS), DRDO, Delhi, India

**Keywords:** *Gingko biloba* L. leaf extract, SK channels, hippocampus, neurodegeneration, memory, hypobaric hypoxia

## Abstract

Hypobaric hypoxia (HH) is a stressful condition, which is more common at high altitudes and can impair cognitive functions. *Ginkgo biloba* L. leaf extract (GBE) is widely used as herbal medicine against different disorders. Its ability to improve cognitive functions, reduce oxidative stress, and promote cell survival makes it a putative therapeutic candidate against HH. The present study has been designed to explore the effect of GBE on HH-induced neurodegeneration and memory impairment as well as possible signaling mechanisms involved. 220–250 gm (approximately 6- to 8-week-old) Sprague Dawley rats were randomly divided into different groups. GBE was orally administered to respective groups at a dose of 100 mg/kg/day throughout the HH exposure, i.e., 14 days. Memory testing was performed followed by hippocampus isolation for further processing of different molecular and morphological parameters related to cognition. The results indicated that GBE ameliorates HH-induced memory impairment and oxidative damage and reduces apoptosis. Moreover, GBE modulates the activity of the small conductance calcium-activated potassium channels, which further reduces glutamate excitotoxicity and apoptosis. The exploration of the downstream signaling pathway demonstrated that GBE administration prevents HH-induced small conductance calcium-activated potassium channel activation, and that initiates pro-survival machinery by activating extracellular signal–regulated kinase (ERK)/calmodulin-dependent protein kinase II (CaMKII) and the cAMP response element–binding protein (CREB) signaling pathway. In summary, the current study demonstrates the beneficial effect of GBE on conditions like HH and provides various therapeutic targets involved in the mechanism of action of GBE-mediated neuroprotection.

## Introduction

Hypobaric hypoxia (HH) at high altitude (HA) has been known to reduce the availability of oxygen to tissue by reducing its partial pressure. Clinical investigations have demonstrated that exposure to acute HH increases susceptibility to high-altitude pulmonary and cerebral edema (HAPE), acute mountain sickness (AMS), and other anomalies such as sleeping disorder, dizziness, and memory loss. Mitochondrial oxygen utilization is essential for ATP generation, which is compromised by the lack of oxygen. Mitochondrial biogenesis affects brain capacity and cognition to some extent by influencing synapse density (Liu et al., 2016). HH causes electrophysiological changes and memory disability in the central nervous system. It is associated with deleterious effects on cognitive functions, neurodegeneration, and state of anxiety and depression (Hale et al., 1996; Basnyat B and Murdoch DR., 2003; Jain et al., 2012, 2013; Kushwah et al., 2016). Neuropsychological functions such as learning and memory, focusing, and processing thoughts are provocatively influenced, which may be, at least partly, because of the episode of neuronal loss in different brain regions. The hippocampus, which plays a significant role in memory function, is one of the brain regions predominantly susceptible to hypoxic damage (Zhao et al., 2019). Owing to the brain's high oxygen expenditure, high iron, high polyunsaturated fatty acid content, and relatively low levels of defense mechanisms against free radicals, it is vulnerable to oxidative stress. HH causes an increase in oxidative stress which involves the generation of both reactive oxygen species (ROS) and reactive nitrogen species (RNS) and is associated with various inflammatory reactions (Droge W., 2002; Maiti et al., 2006). Brain hypoxia along with different medical conditions results in apoptosis *via* caspase-3 activation in the hippocampus and other brain regions (Maiti et al., 2008; Kushwah et al., 2018).

*Ginkgo biloba* L., a well-established medicinal plant, has been reported to have several beneficial effects that include antioxidant potential in reducing the oxidative stress, neurodegeneration, and memory impairments (Blecharz et al., 2009; Zhao et al., 2012) caused by hypoxia. Hence, *Ginkgo biloba* L. leaf extract (GBE) may reduce the deleterious effects observed during hypoxia exposure. GBE also designated as ‘‘EGb 761’’ contains around 30 kinds of flavonoids and their derivatives and terpenoids such as ginkgolide A, ginkgolide B, ginkgolide C, and bilobalide (Zhu et al., 1998; Shinozuka et al., 2002). GBE also promotes episodic memory function in patients with mild cognitive impairment (Martin et al., 2011; Mazumder et al., 2017). *Ginkgo* has been reported to have a scavenging effect as established by the reduction of malondialdehyde (MDA) and glutathione (GSH) in the brainstem and the cerebellum (Chandrasekaran et al., 2002). GBE can also counteract apoptosis, increase neuroplasticity, and attenuate neuronal death in cases of global ischemia and glutamate-induced excitotoxicity (Belviranl M and Okudan N., 2015). Additionally, GBE supplementation has been suggested to increase brain-derived neurotrophic factor (BDNF) expression in several brain regions (Hammond et al., 2006).

We have previously established that the hypoxia-induced learning and memory impairment were improved by blocking small conductance calcium-activated potassium channels (SK channels) with apamin (Kushwah et al., 2018). These channels are widely distributed in peripheral and central nervous systems. The threshold for the stimulation of hippocampal synaptic plasticity could be altered *via* SK2 channels by regulation of calcium concentrations, and that modulates excitatory postsynaptic potentials (EPSPs) essential for the induction of long-term potentiation (LTP) (Lin et al., 2008; Kuiper et al., 2012). These channels modulate synaptic plasticity; hence, SK2 channel inhibition improves learning (Vick et al., 2010). In contrast, increasing SK channel activity impairs learning (Li et al., 2001; Adelman et al., 2012). The study indicates that GBE increases nitric oxide synthase (NOS) activity by means of SK channels in cultured porcine endothelial cells (LeBel et al., 1990), further supporting the view that GBE modulates the activity of SK channels.

Thus, the present study was designed to investigate the effect of GBE on hypoxia-induced memory impairment and neurodegeneration *via* inhibition of SK2 channels and activation of extracellular signal–regulated kinase (ERK)/calmodulin-dependent protein kinase II (CaMKII) and the cAMP response element–binding protein (CREB) signaling pathway.

## Materials and Methods

### Chemicals and Reagents

All chemicals used to perform this study were procured from Sigma-Aldrich (St. Louis, United States). Primary antibodies for CREB, CaMKII, ERK, SK2, BDNF, active caspase-3, and beta-actin were procured from Abcam (Cambridge, United States). Anti-rabbit and goat secondary antibodies were procured from Millipore (Darmstadt, Germany). Chemicals to perform immunoblotting were procured from Bio-Rad (California, United States). Kits for ROS, glutathione/glutathione disulfide (GSH/GSSG), and total antioxidant capacity were purchased from BioAssay Systems (CA, United States). GBE was purchased from Ambe Pharmaceuticals Pvt. Ltd. (India). Thin-layer chromatography (TLC) Silica gel 60 F_254_ was purchased from Merck (Darmstadt, Germany), and solvents used for high-performance TLC (HPTLC) were of analytical grade.

### Animals

Male Sprague Dawley (SD) rats of 220–250 g (approximately 6–8 weeks old) were utilized for this study. A total of 75 animals were used in the study; the animals were preserved in the experimental animal house facility of our institute maintaining day and night cycles of 12 h each. The temperature was maintained at 25 ± 2°C, and the humidity was kept 60 ± 5%. Food and water were available *ad libitum*. All experiments performed were approved by the Committee for the Purpose of Control and Supervision of Experiments on Animals (CPCSEA), Government of India. The Institutional Committee for Animal Care and Use (ICACU) of the Defense Institute of Physiology and Allied Sciences approved the experimental and animal care protocol for this study (27/40/RBi/SL/99/CPCSEA).

### Hypobaric Hypoxia Simulation

**FIGURE 1:**
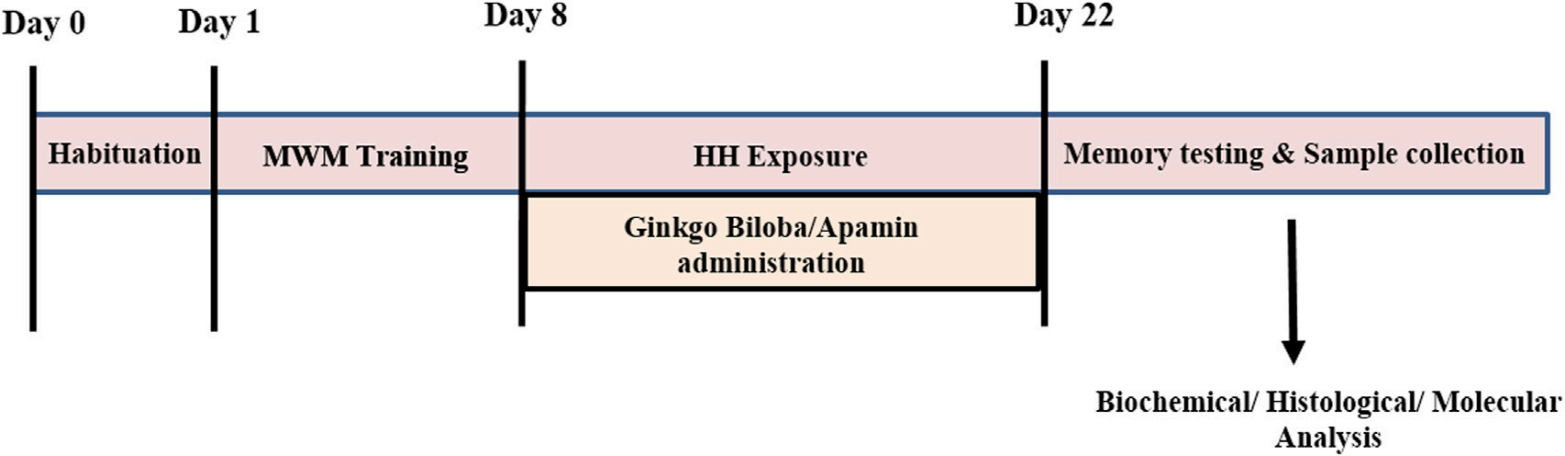
Timeline diagram showing the experimental design of the study. Day 0 represents habituation of the rats in the MWM tank followed by 8 days of training. At day 8, rats were exposed to HH at 25,000 ft for 14 days along with GBE and apamin treatment. After completion of exposure, memory testing and sample collection were performed for further experiments.

Animals were divided into five groups, i.e., control, control + GBE, HH alone, HH + GBE, and HH + apamin. HH-related groups were exposed in an animal decompression chamber for continuous 14 days simulated equivalent to an altitude of 7,600 m (25,000 ft). Control and control + GBE groups were maintained in conditions of normal atmospheric pressure. The animals were brought down to the sea level for 15 min every day for replacement of food and water. Figure 1 presents the schematic timeline of the experimental design in the study.

#### High-Performance Thin Layer Chromatography Profiling and Estimation of Quercetin

GBE contained 24.80% *Ginkgo* flavone glycosides and 6.08% terpene lactones. 20 mg GBE was dissolved in 1 ml methanol. The content was vortexed for 5 min for proper dissolution and then centrifuged at 1,000 rpm (67 xg). The supernatant was collected, and the sample was analyzed for phytochemical analysis with its suitable marker(s) using HPTLC densitometry analysis (Mukherjee et al., 2008). HPTLC profiling of the samples was performed using a CAMAG Linomat 5 sample applicator (CAMAG, Switzerland). In brief, 10 μL from sample was applied in doublet with 8 mm wide band length to pre-washed and activated Silica gel 60 F_254_ pre-coated HPTLC plates with the nitrogen flow providing a delivery speed of 150 nL/s. The TLC plate was developed in a pre-saturated TLC development chamber containing toluene: ethyl acetate: glacial acetic acid (6:3:1, v/v/v) as a solvent system. The plates were developed to a distance of 8.0 cm at room temperature (25°C). After drying, the spots on the developed plates were visualized under visible (white), short UV (254 nm), and long UV (366 nm) light and scanned at 254 and 366 nm (Anjum et al., 2017). HPTLC profiling of the sample was performed after the successive extraction of the different drugs. Thereafter, HPTLC densitometry analysis was performed using its suitable markers at 254 and 366 nm. The resulting data reveal that quercetin was found as a major constituent at retention factor (Rf) 0.36.

#### Drug Administration

GBE was dissolved in sterile distilled water and was orally administered to respective groups at a dose of 100 mg/kg/day (Wahab et al., 2012). Apamin (0.5 ng, *i.c.v.*) was used to inhibit the SK2 channels and was given stereotaxically as per the protocol mentioned in our previous study (Kushwah et al., 2018). Briefly, after anesthetizing the animals, a minor hole was drilled into the skull by which a guide cannula (OD, 0.55 mm; ID, 0.31 mm) was then inserted in the right lateral cerebral ventricle with subsequent coordinates: 0.7 mm from bregma, 1.7 mm lateral to midline, and 4.0 mm from dura. Using the cannula, apamin (0.5 ng/day) was infused into the ventricles for continuous 14 days during HH exposure. Experimental groups are shown in Table 1.

**TABLE 1:** Experimental groups.

S. No.	Groups	HH exposure	Interventions
1	Control	Nil	Nil
2	Control + GBE	Nil	GBE (100 mg/kg), 14 days
3	HH	25,000 ft/14 days	Nil
4	HH + GBE	25,000 ft/14 days	GBE (100 mg/kg), 14 days
5	HH + apamin	25,000 ft/14 days	Apamin (SK2 inhibitor) 0.5 ng, *i.c.v.*

### Sample Preparation

#### Sample for Morphological Analysis

Samples for morphological analysis were prepared as per the previously standardized protocol. Briefly, after perfusion with PBS and fixation in 4% paraformaldehyde (PFA) solution, the entire brain was removed in sterile conditions and post-fixed in PFA for 24 h. Dehydration was done in 10, 20, and 30% sucrose solution for 24 h. Brain tissues were embedded in cryo fluid and kept in the cryostat chamber. 30 µm thick sections were cut in the cryostat (Leica 3050, Germany). For morphological study, cresyl violet and Fluoro-Jade B staining was performed. Immunohistochemistry (IHC) of active caspase-3 was performed as per the standardized protocol (Kushwah et al., 2016, 2018). Briefly, sections were subjected to sodium citrate treatment at 100°C for 10 min for antigen retrieval, followed by washing in PBST (0.1% Triton) 3 × 10 times. Sections were permeabilized in 0.25% PBST and washed in PBST 3 × 10 times. Active caspase-3 (1:250) in 5% normal goat serum was added into each section for 48 h at 4°C. After washing with PBST, respective secondary antibody (1:500) was added for 2 h at room temperature, followed by washing again with PBST 3 × 10 times, and sections were developed in the DAB solution. Sections were further subjected to 50, 70, and 100% alcohol treatment and treated with xylene and later mounted in a DPX mounting medium and viewed under the microscope**.**

#### Sample for Immunoblotting

Immunoblotting was performed as per the previously published protocol. Briefly, membranes were incubated with primary antibodies CREB (1:1,000), phospho-CREB (p-CREB) (Ser133) (1:500), ERK (1:1,000), phospho-ERK (p-ERK) (Thr202/Thr204) (1:500), CaMKII (1:500), phospho-CaMKII (p-CaMKII) (Thr305) (1:250), BDNF (1:1,000), beta-actin (1:5,000), and active caspase-3 (1:500). After washing, membranes were further incubated with HRP-conjugated anti-rabbit and goat secondary antibodies (1:10,000) for 2 h and then developed through a chemiluminescence peroxidase kit (Sigma, St. Louis, United States). The protein expression in each group was quantified by densitometry analysis (Kushwah et al., 2016, 2018).

### Memory Testing

The spatial reference memory task was performed using the Morris water maze (MWM). Animals were randomly divided into different groups and were tested for anxiety and depression before memory testing as per the previously described protocol (Jain et al., 2013). Briefly, the rats were habituated in the water tank for 1 day followed by 8 days of training to reach the platform. After completion of training, memory tests and probe trials were performed.

### Biochemical Estimations

Following HH exposure, all animals were sacrificed, and the hippocampi were removed at 4°C in ice-cold PBS. Homogenization was carried out in 0.15 M KCl to obtain a 10% homogenate. The homogenate was then centrifuged at 10,000 rpm (6,708 xg) for 10 min, and the clear supernatant obtained was used for estimating biochemical parameters (Hota et al., 2008).

#### Glutathione/GSSG Measurement

The assay was performed as per the manufacturer’s protocol using BioAssay Systems' GSH/GSSG Assay Kit. Briefly, cell lysate for GSSG and total glutathione was prepared using a specific cell lysis buffer. Deproteination was performed using metaphosphoric acid (MPA) reagent. The OD was taken at 412 nm at 0 min and again at 10 min.

#### Reactive Oxygen Species Estimation

Free radicals were estimated spectrofluorimetrically by 2′,7′-dichlorofluorescein-diacetate (DCFHDA) as per the standardized protocol of LeBel et al. (1990). Briefly, 1.494 ml of 0.1 M PBS (pH 7.4) was added to 25 µL of the sample, and then 6 µL of DCFHDA (1.25 mM) was added. After that, incubation was done for 15 min at 37°C in the dark, and fluorescence was measured at 488 nm excitation and 525 nm emission. The values obtained were transformed to fluorescent units per milligram of protein by calculating the protein found in 25 µL of the respective sample from a standard curve (Hota et al., 2008).

#### Lipid Peroxidation

The measurement of lipid peroxidation was done spectrophotometrically by calculating MDA formed as a product, as designated by Utley et al. (1967). A respective molecule of malondialdehyde reacts with two molecules of thiobarbituric acid (TBA) to produce a colored MDA–TBA complex that can be further measured spectrophotometrically at 531 nm (Hota et al., 2008).

#### Lactate Dehydrogenase Activity

LDH activity in the hippocampus tissue was estimated using the LDH assay kit from RANDOX (RANDOX Laboratory Ltd., United Kingdom). The assay was performed as per the protocol suggested by the manufacturer with minor modifications. Briefly, the assay was carried out by adding 10 μL of sample to 250 μL of the reconstituted reagent containing pyruvate and NADH in phosphate buffer. Phosphate buffer was added as a blank. The absorbance was then taken at 340 nm in an enzyme-linked immunosorbent assay (ELISA) reader after 30 s that was considered at 0 min and then after 1, 2, and 3 min from the initial reading. The result thus obtained was expressed in percentage taking the mean control value to be 100%.

#### Total Antioxidant Capacity Assay

The assay was performed according to the manufacturer’s instructions (BioAssay Systems). The sample and standard were prepared as per the protocol, and the plate was thoroughly mixed and incubated for 10 min at room temperature. The OD was taken at 570 nm on a Synergy H4 hybrid reader (BioTek).

#### Glutamate Assay

The glutamate assay was performed by the assay kit by Abcam (Cambridge, United States) as per the manufacturer’s instruction. Briefly, the glutamate enzyme mix and glutamate developer were solubilized; standards and samples were prepared as per the instructions. 100 µL of reaction mix was added to the standard and sample wells. 100 µL background reaction mix was added to background sample wells. The plate was incubated at 37°C for 30 min protected from light. The OD of the plate was taken at 450 nm.

### Morphological Staining

#### Fluoro-Jade B Staining

For neurodegeneration study, the brain sections were stained with Fluoro-Jade B, a polyanionic fluorescence derivative that selectively binds to degenerating neurons. Staining was carried out as per the standard protocol (Kushwah et al., 2018). The samples were viewed under the fluorescence microscope (Olympus, Japan) by means of the FITC filter. A positive green fluorescence signal designates degenerative neurons.

#### Cresyl Violet Staining

Morphological alterations were observed by CV staining. CV is commonly utilized to stain Nissl material in the cytoplasm of neurons in PFA- or formalin-fixed tissue. This was performed as per our previously published protocol (Kushwah et al., 2018) to check the pyknotic neurons.

### Statistical Analysis

MWM data were analyzed using the ANY-maze software. For the behavioral study, 7–10 rats in each group were taken. Histology data (IHC, Fluoro-Jade B–positive cells, and pyknotic cells) were investigated *via* ImageJ software. Briefly, images were taken of the whole hippocampus from six different rats, and six random sections were taken from each rat and mounted on two slides (thus, each slide comprises three sections from each experimental group). For neutral investigation, several images of diverse regions of the hippocampus were taken from each section at ×20 magnification. Out of three sections from each slide, nine random images were occupied, and thus, a total of 18 random images were taken from two slides, i.e., each group. Densitometry analysis was performed for all immunoblots. Data for all experimentations were articulated as mean ± SEM. Non-parametric one-way ANOVA and Bonferroni’s multiple correction tests were utilized for multiple intergroup comparisons. Statistical investigations were performed by GraphPad Prism 5 throughout the study unless specified otherwise. The significance value for all tests was set at *p* < 0.05 (Kushwah et al., 2018).

## Results

The study has been conducted to explore the effect of GBE on HH-induced damage at behavioral and molecular levels. Further mechanisms of action were also explored. GBE comprises different *Ginkgo* flavone glycosides, and we estimated quercetin using HPTLC (Supplementary Figures 1A,B). The analysis showed quercetin was found as a major constituent at Rf 0.36. The content of quercetin in GBE was found as 60.74 ± 0.0629 µg/mg, w/w, at 254 nm.

### *Ginkgo biloba* L. Leaf Extract Prevents Hypobaric Hypoxia–Induced Spatial Memory Impairment

**FIGURE 2:**
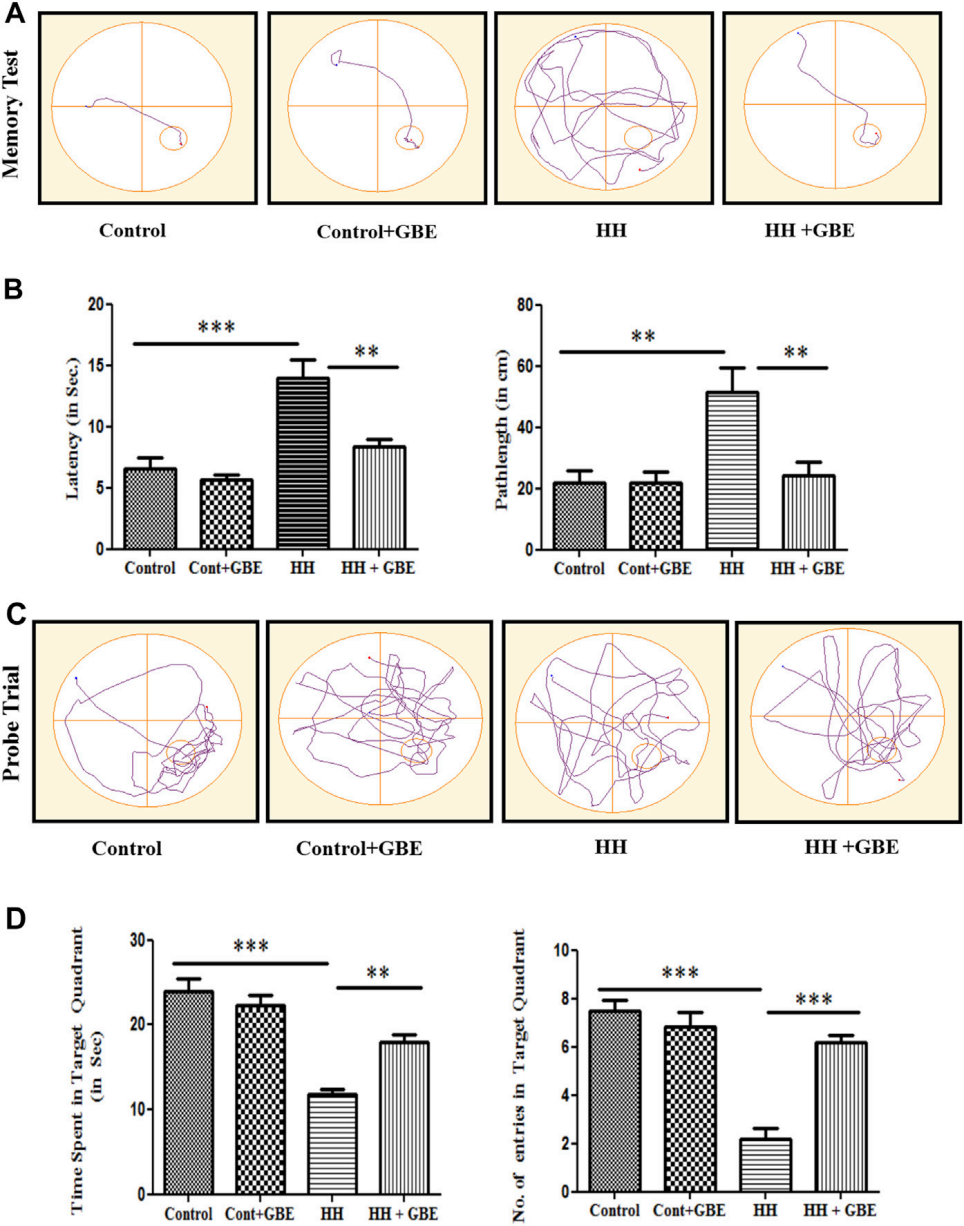
GBE improves the spatial memory performance of rats in the MWM. The effect of GBE on memory was studied using the MWM spatial memory test. Representative track plots of the memory test and probe trial of control, control + GBE, HH, and HH + GBE groups **(A, C)**. Treatment of rats with GBE showed protective effect on HH-induced memory impairment as evident from the decreased latency and path length **(B)** and increased time spent and number of entries in the target quadrant **(D)** as compared to the HH group. One-way ANOVA with the Bonferroni test was used to analyze the data. Data are represented as mean ± SEM. “**” represents *p* < 0.01, whereas “***” represents *p* < 0.001.

HH-mediated memory impairment has been well studied in various studies (Shukitt-Hale et al., 1994; Jayalakshmi et al., 2007; Kushwah et al., 2018). A similar result was observed in the present study which shows a significant increase in latency to reach the platform and path length after 14 days of HH exposure (Figures 2A,B). Also, during the probe trial, the time spent and the number of entries in the target zone were decreased significantly (*p* < 0.001) after 14 days of HH exposure (Figures 2C,D). On the contrary, GBE administration ameliorated these effects by increasing the time spent (*p* < 0.01) and number of entries in the target quadrant (*p* < 0.001) (Figures 2C,D). Also, the latency and path length to reach the platform were significantly decreased (*p* < 0.01) in the group administered GBE, during 14 days of HH exposure (Figures 2A,B).

### *Ginkgo biloba* L. Leaf Extract Ameliorates Hypobaric Hypoxia–Induced Oxidative Stress

**FIGURE 3:**
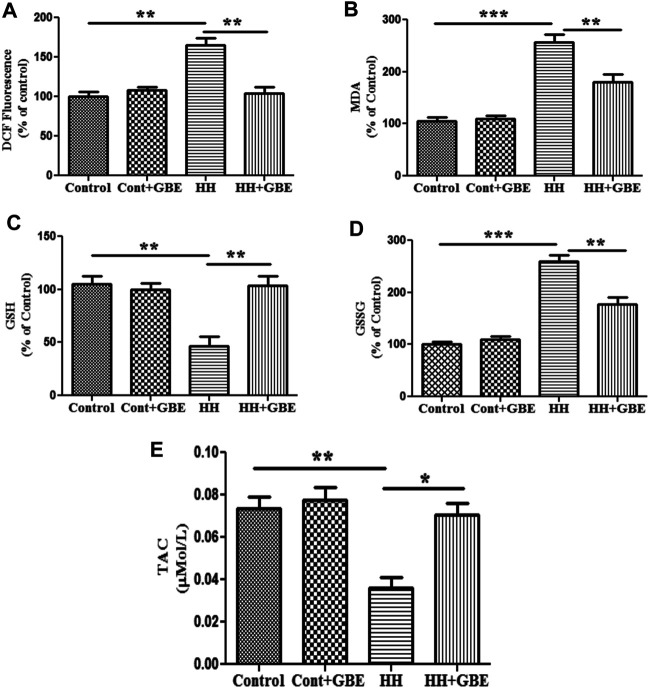
GBE prevents HH-induced oxidative damage. Representative bar graphs of oxidative stress markers such as ROS level **(A)**, lipid peroxidation (MDA) **(B)**, GSH **(C)**, GSSG **(D)**, and TAC **(E)**. 14 days of HH exposure increased the level of ROS and MDA; however, GBE significantly decreased their elevation. GSH was decreased after 14 days of HH exposure, and GBE treatment significantly reverted these effects. HH increased the level of GSSG, which was further reduced by GBE treatment. Furthermore, TAC was increased significantly (*p* < 0.05) after GBE treatment as compared to the HH group. One-way ANOVA with the Bonferroni test was used to analyze the data. The results are expressed as mean ± SEM. *p < 0.05, **p < 0.01, and ***p < 0.001.

Oxidative stress is known to be one of the major causes of HH-induced cognitive deficit. Hence, we further explored the effect of GBE on oxidative stress markers and apoptosis. Free radical generation during oxidative stress was measured as ROS level estimation by the DCFDA method. The elevated ROS generation is a direct measure of oxidative stress which was observed in the present study as evident from the increased (*p* < 0.01) ROS level in groups exposed to HH. On the contrary, GBE significantly decreased (*p* < 0.01) the production of ROS in the hippocampus as compared to the 14-day HH group (Figure 3A).

Furthermore, we also studied lipid peroxidation *via* estimating the MDA level. Like ROS, HH exposure for 14 days significantly increased the level of MDA in hippocampal tissue (*p* < 0.001) as an indicator of lipid peroxidation. However, the group treated with GBE along with 14 days of HH exposure had a reduced level (*p* < 0.01) of MDA in hippocampal tissue when compared to the group exposed to HH (Figure 3B).

Antioxidant status was also studied *via* measuring the intracellular GSH level, which is an important component of the cellular antioxidant defense system and expressed in cells in two different states, i.e., GSH (reduced) and GSSG (oxidized). The present study shows that the level of GSH decreased significantly (*p* < 0.001) and GSSG was significantly increased (*p* < 0.001) after 14 days of HH exposure, whereas GBE treatment reversed this effect by restoring the antioxidant status of the cell in the hippocampus as evident from the increased (*p* < 0.001) GSH level and reduced (*p* < 0.001) GSSG level **(** Figures 3C,D **)**.

In addition to the GSH/GSSG level, total antioxidant capacity (TAC) was also analyzed and similar findings were observed as TAC was significantly reduced by 14 days of HH exposure which was further ameliorated (*p* < 0.05) by GBE treatment (Figure 3E).

### *Ginkgo biloba* L. Extract Precludes Hypobaric Hypoxia–Induced Neurodegeneration by Reducing Apoptosis

**FIGURE 4:**
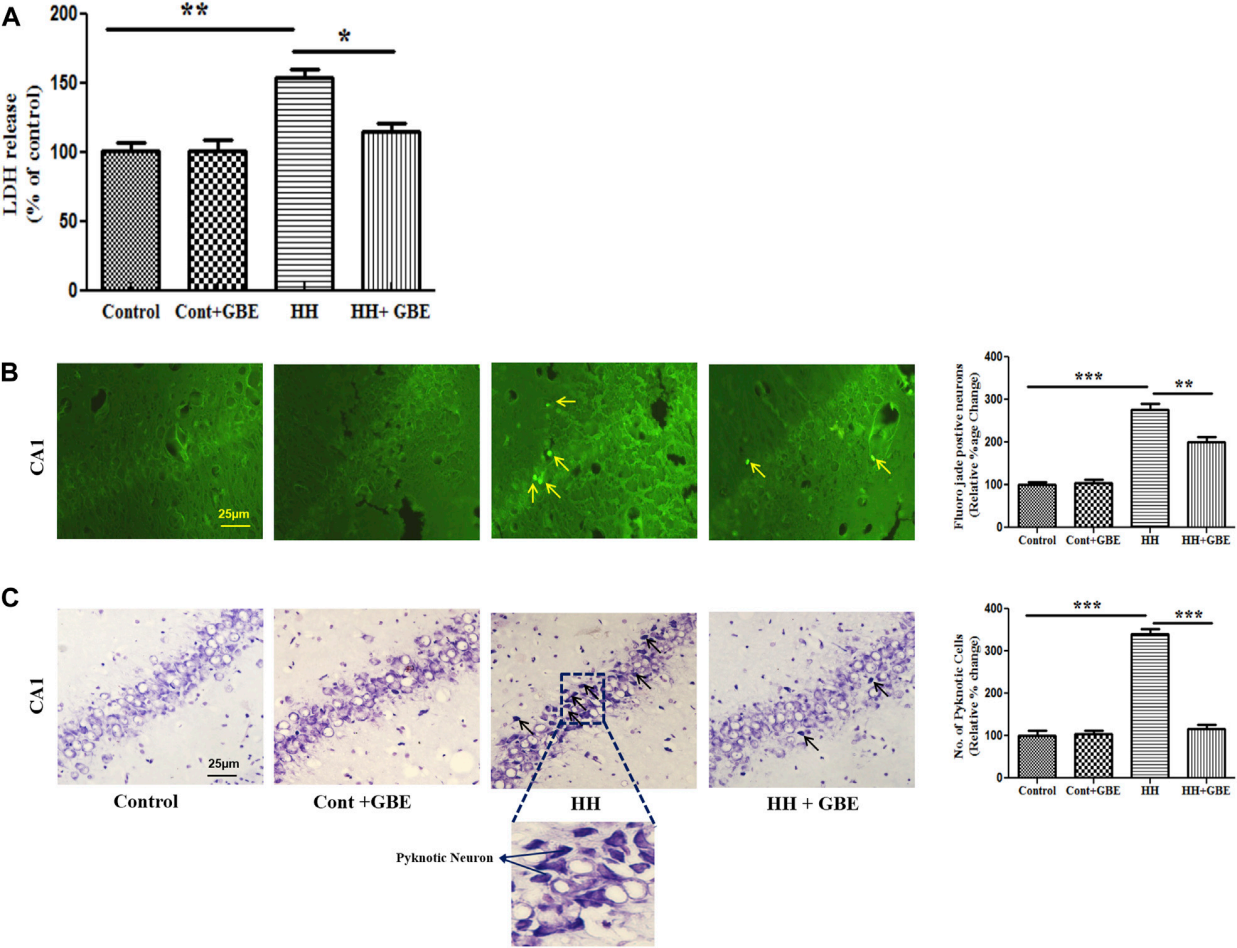
GBE prevents LDH leakage and ameliorates neuronal damage and morphological alterations during HH exposure. Bar graph of LDH **(A)** that shows decreased LDH release after GBE treatment which was increased in HH exposure. HH leads to neurodegeneration as evident from the increased number of Fluoro-Jade B–positive cells in the hippocampus. Images and bar graph show an increase in Fluoro-Jade B–positive cells in 14 days of HH exposure as compared to the GBE-treated group which significantly reduced the Fluoro-Jade B–positive cells **(B)**. Cresyl violet staining was done to assess pyknotic cells which were higher in the HH group, whereas GBE significantly decreased the pyknotic cell count when compared to the HH group **(C)**. Black arrows indicate pyknotic cells. One-way ANOVA with the Bonferroni test was used to analyze the data. Data are represented as mean ± SEM. *p < 0.05, **p < 0.01, and ***p < 0.001. The scale bar represents 25 µm.

Neurodegeneration or cell death was studied at different levels and approaches, i.e., biochemically, morphologically, and at the molecular level. Initially, cell death was studied biochemically by estimating lactate dehydrogenase (LDH) levels in different groups. It was found that 14 days of HH exposure significantly increased the LDH level (*p* < 0.001) which indicates elevated cell death when compared to the control group. GBE administration reduced the LDH level to a significant level and hence reduced cell death (Figure 4A).

To study neurodegeneration, Fluoro-Jade B staining was performed, and it was found that 14 days of HH exposure significantly (*p* < 0.001) increased Fluoro-Jade B–positive cells in the hippocampus in comparison with the control group. However, GBE administration significantly reduced (*p* < 0.01) the number of positive cells (Figure 4B). Similarly, cresyl violet staining also showed that GBE significantly (*p* < 0.0001) reduced the total number of pyknotic cells as compared to the HH group (Figure 4C).

**FIGURE 5:**
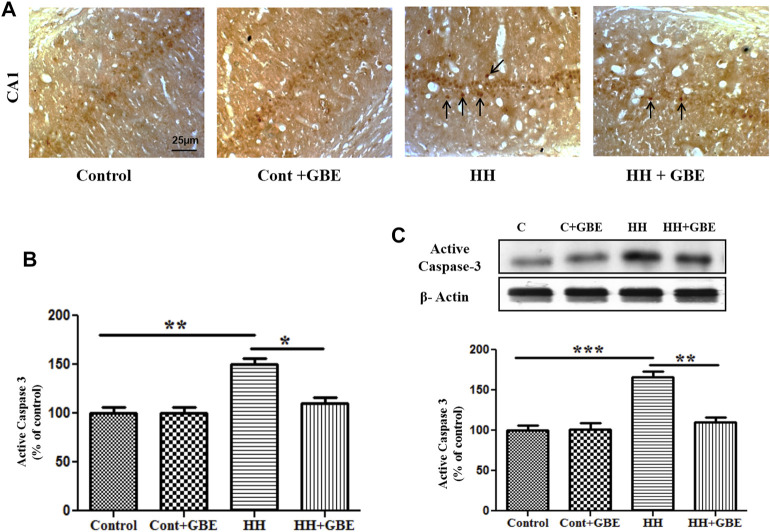
GBE reduces HH-induced apoptosis in the hippocampus. Images show the caspase-3–positive cells in different groups **(A)**. Graphical representation indicates the increased number of caspase-3–positive cells in 14 days of HH exposure, whereas GBE significantly reduced the caspase-3–positive cells when compared to the HH group **(B)**. Black arrows indicate activated caspase-3–positive cells. Immunoblot results also indicate reduced expression of active caspase-3 in groups treated with GBE when compared to the HH group **(C)**. One-way ANOVA with the Bonferroni test was used to analyze the data. Data are represented as mean ± SEM. *p < 0.05, **p < 0.01, and ***p < 0.001. The scale bar represents 25 µm.

Apoptosis is one of the major processes leading to neurodegeneration during HH exposure. Further activated caspase-3 expression, an apoptotic marker, was examined. The caspase-3 antibody used in this study is used to detect the active/cleaved form of caspase-3 after apoptosis induction. Immunohistochemistry showed increased apoptosis in the hippocampus at 14 days of HH exposure as evident from the increased number of activated caspase-3–positive cells in the HH group. However, GBE prevents apoptosis shown by the reduced (*p* < 0.05) number of activated caspase-3–positive cells (Figures 5A,B). Likewise, immunoblot results followed a similar pattern and showed reduced (*p* < 0.01) expression of activated caspase-3 at the molecular level in the group treated with GBE when compared to the HH alone group (Figure 5C).

### *Ginkgo biloba* L. Leaf Extract Promotes Neuroprotection *via* SK2 Inhibition

**FIGURE 6:**
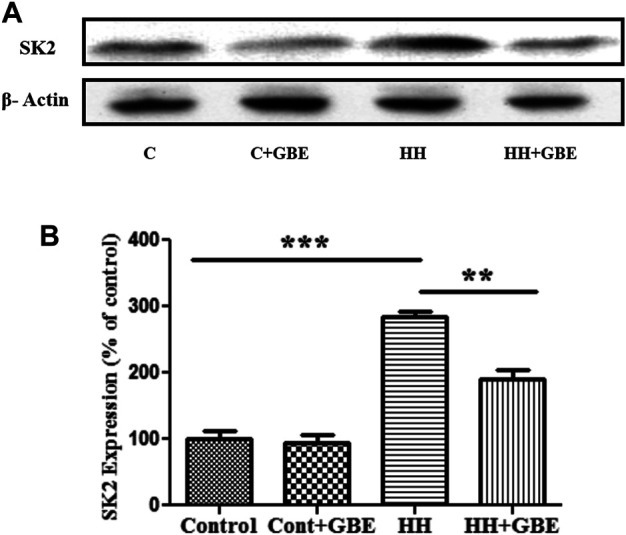
GBE reduces expression of the SK2 channel. Immunoblot indicates expression of the SK2 channel **(A)**. The optical density analysis of SK2 expression using ImageJ software showed that, after 14 days of HH exposure, SK2 expression increased, while GBE significantly reduced its expression **(B)**. One-way ANOVA with the Bonferroni test was used to analyze the data. Data are represented as mean ± SEM. **p < 0.01 and ***p < 0.001.

Previously, we have shown increased activity and expression of SK2 channels as one of the causes for HH-induced deterioration (Kushwah et al., 2018). The expression of SK2 channels significantly reduced in groups administered GBE (Figures 6A,B) when compared to a group exposed to HH alone for 14 days. Also, after estimating the glutamate level in different groups, it was observed that 14 days of HH exposure caused glutamate excitotoxicity as evident from the increased glutamate level (Figure 6A). On the contrary, the glutamate level significantly reduced in the GBE-treated group in comparison with the HH alone group.

**FIGURE 7:**
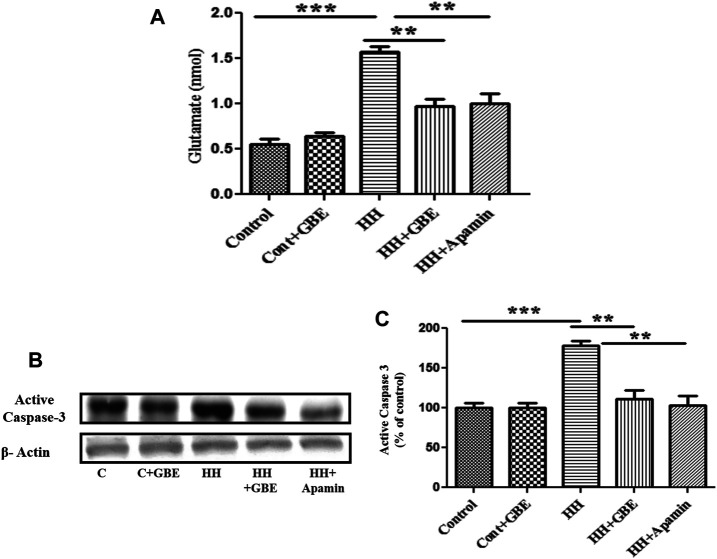
GBE reduces glutamate excitotoxicity and activates caspase-3 expression *via* SK2 inhibition. The graphic representation shows that the glutamate level increased on the 14th day of HH exposure, and the glutamate level decreased significantly after GBE treatment. However, compared with the HH group, the SK2 inhibitor, apamin, can also reduce the toxicity of glutamate **(A)**. Immunoblotting showed that compared with the control, the expression of activated caspase-3 increased significantly after 14 days of exposure to HH, while the GBE and SK2 inhibitor, apamin, significantly reduces its expression **(B)**. Densitometry analysis of the activated caspase-3 expression using ImageJ software **(C)**. One-way ANOVA with the Bonferroni test was used to analyze the data. Data are represented as mean ± SEM. **p < 0.01 and ***p < 0.001.

Furthermore, to validate the role of GBE in SK2 inhibition, a comparison was made with chemical inhibition of SK2 *via* apamin. Glutamate and apoptosis markers were studied, and it was found that the GBE-treated group reduces glutamate excitotoxicity (Figure 7A) and prevents apoptosis (Figures 7B,C) comparable to a group administered apamin during HH exposure. This indicates GBE is working through inhibition of SK2 channels during chronic HH exposure for 14 days.

### SK2 Inhibition–Mediated Neuroprotection by *Ginkgo biloba* L. Leaf Extract Involves Calmodulin-Dependent Protein Kinase II/Extracellular Signal–Regulated Kinase/cAMP Response Element–Binding Protein Pathway

GBE is known to have a multifactorial response. In the present study too, it leads to SK2 inhibition which further inhibits cellular death machinery by modulating downstream signaling as well as activating the survival pathway.

**FIGURE 8:**
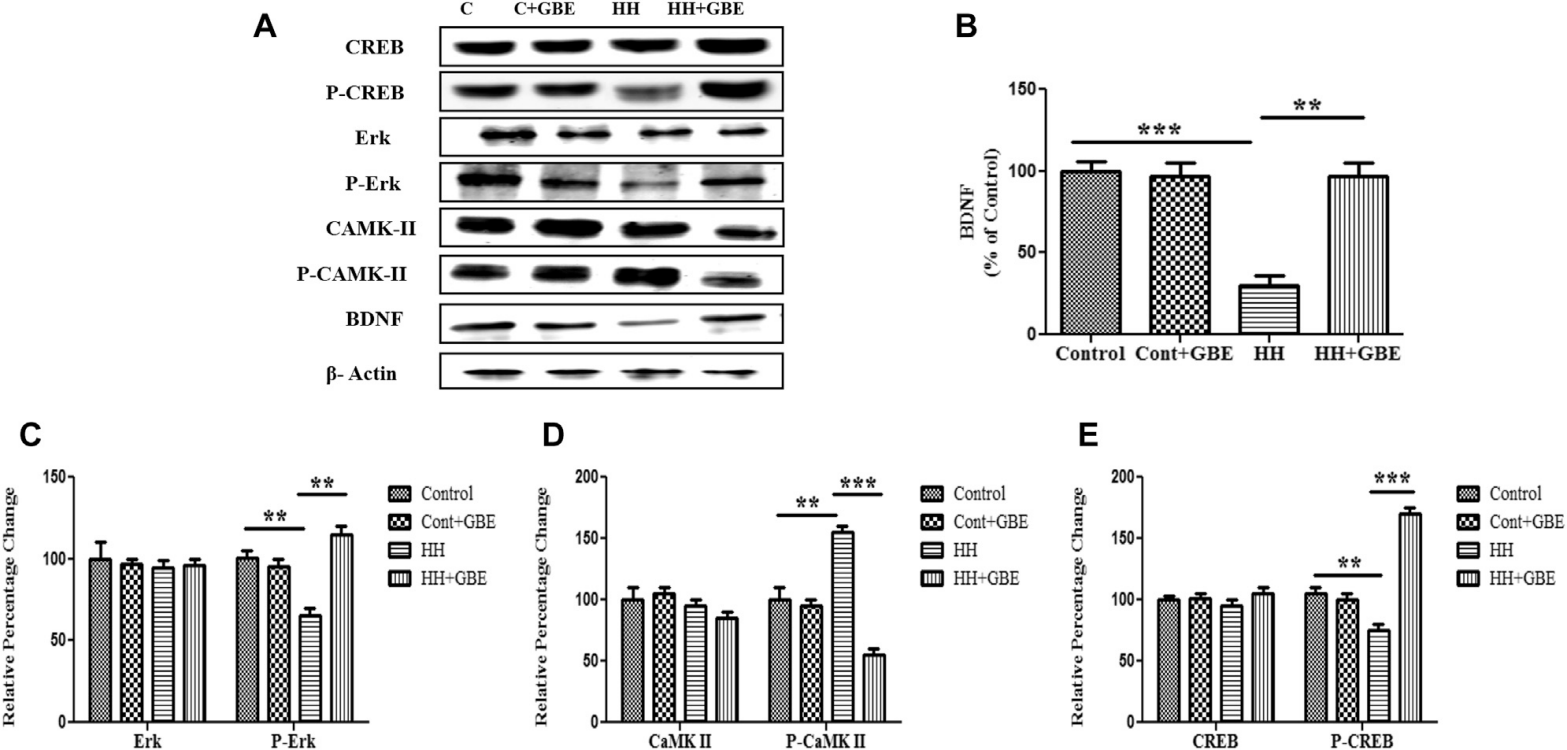
GBE activates the ERK/CaMKII/CREB pathway. Immunoblot of CREB, p-CREB, ERK, p-ERK, CaMKII, p-CaMKII, and BDNF proteins **(A)**. Further densitometry analysis of respective immunoblots was performed through ImageJ software and represented as a bar graph of BDNF **(B)** p-ERK **(C)**, p-CaMKII **(D)**, and p-CREB **(E)**. No significant changes were observed in ERK, CaMKII, and CREB expressions. One-way ANOVA with the Bonferroni test was used to analyze the data. Data are represented as mean ± SEM. *p < 0.05, **p < 0.01, and ***p < 0.001.

**FIGURE 9:**
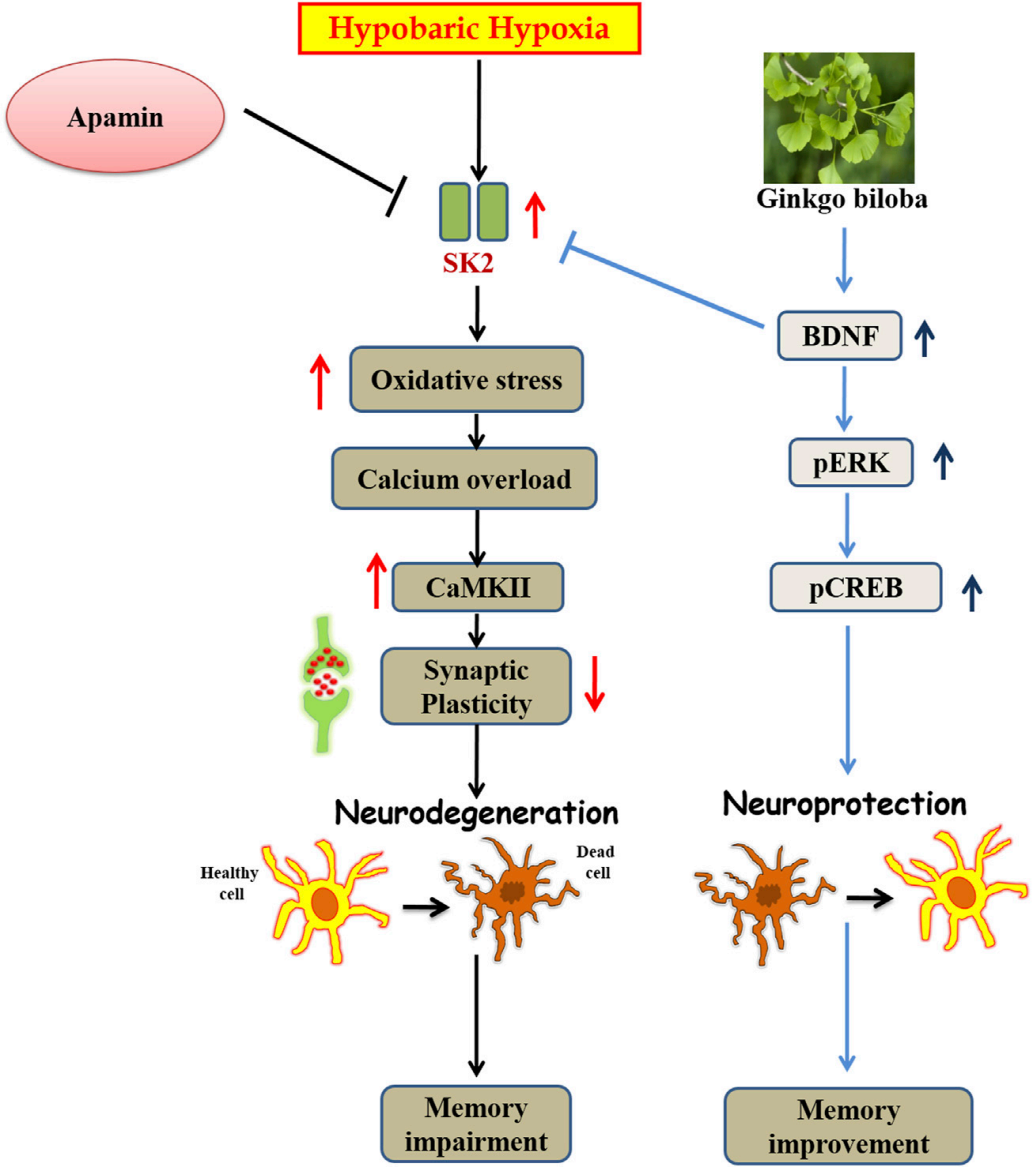
Graphical representation of abstract: Schematic diagram representing the mechanism of action of *Ginkgo biloba* L. during HH.

BDNF is well known to promote cell survival and neuroprotection. BDNF reduces the SK2 component by phosphorylation, causes its inactivation, and strengthens synaptic transmission (Figure 8A). We also observed restoration of the depleted BDNF level after GBE treatment during 14 days of HH exposure (Figure 8B). The ERK/mitogen-activated protein kinase (MAPK) pathway has been exhibited to play a vital role in anti-apoptotic mechanisms. Hence, we found it interesting to explore the effect of GBE treatment on ERK activation in the hippocampus during HH exposure. As shown in Figure 8C, 14 days of HH exposure significantly reduces the ERK activation and, hence, phosphorylation which is further ameliorated by GBE treatment. Activation of the ERK pathway is known to be modulated by the upstream signaling molecule—the Ca^2+^-sensitive calcium-modulated kinases (CaMKs). We have also studied the expression of CaMKII, and it was found that GBE treatment reduces the activity of CaMKII, which indicates that calcium homeostasis is maintained, thus promoting neuroprotection (Figure 8D). Since ERK further activates its downstream transcription factor, i.e., cAMP response element–binding protein (CREB), which plays an important role in cell survival, we next studied the phosphorylation of CREB after GBE treatment and speculated that administering GBE during HH exposure can restore the phosphorylation level of CREB that was only reduced during HH, so that the basal level of CREB in different groups remains the same (Figures 8E, 9).

## Discussion

The present study was aimed to investigate the efficacy of GBE on HH-induced memory impairment and neurodegeneration in rats. Chronic HH exposure is well documented for memory impairment and neuronal loss in different brain regions especially the hippocampus (Maiti et al., 2008) which can be attributed to a variety of factors such as oxidative stress, glutamate excitotoxicity, calcium overload, and synaptic dysfunctions.

HH conditions have shown detrimental effects on spatial memory (Jain et al., 2013). The hippocampus is one of the key brain regions involved in spatial memory formation. Different durations of HH exposure at different altitudes showed significant impairment in spatial memory which got worse in chronic HH exposure conditions (Shukitt et al., 1994; Muthuraju and Pati S., 2014). Similarly, our study also found spatial memory impairment in the MWM test during chronic exposure to HH for 14 days as evident from the increased latency and path length to reach the platform and decreased time spent in the targeted quadrant and number of platform crossing. On the contrary, GBE that has different beneficial ingredients for mental health showed amelioration in detrimental effect of chronic HH exposure on spatial memory. Similar observations were reported previously by Vaghef et al. (2017) which illustrated protective effects of *Ginkgo biloba* L. on oxidative stress as well as memory impairments induced by transient cerebral ischemia. GBE has been reported to exhibit anti-inflammatory and neuroprotective properties in other studies (Liang et al., 2018).

Oxidative stress is found to be involved in the pathogenesis of different neurological disorders such as arteriosclerosis, amyotrophic lateral sclerosis, Parkinson’s disease, and Alzheimer’s disease (Belviranl M and Okudan N., 2015; Zhou et al., 2017). HH causes cellular oxidative damage with consequent damage to lipids, proteins, and DNA that could result in cognitive deficit (Maiti et al., 2006; Jayalakshmi et al., 2007; Hota et al., 2008). In agreement with this, we also observed major increment in oxidative stress parameters such as ROS, lipid peroxidation, and imbalance in antioxidant status (GSH/GSSG level) with chronic exposure to HH. Comparable to our study, other studies also substantiate antioxidant efficacy of GBE by reducing oxidative stress and enhancing the antioxidant level in different neurological disorders (Lugasi et al., 1999; Naik et al., 2006; Aydin et al., 2016; Achete et al., 2020). *Ginkgo biloba* L. has different active ingredients that account for its antioxidant property.

We further examined neurodegeneration in different groups that shows that GBE expressively reduced the HH-mediated neuronal loss as apparent from the decreased number of Fluoro-Jade B–positive neurons and activated caspase-3–positive neurons, which also augmented after 14 days of HH exposure in the hippocampal tissue. HH exposure also altered the neuronal morphology as evident from cresyl violet staining; however, administration of GBE resulted in recovery of the neuronal morphology by reducing the number of pyknotic cells in the GBE-treated group; similar findings were observed in another study also (Kumari et al., 2020). One of the active ingredients of GBE, i.e., ginkgetin (a natural biflavonoid isolated from leaves of *Ginkgo biloba* L.), significantly represses cell apoptosis brought through the caspase-3 and the Bcl2/Bax pathway (Wang et al., 2016) and averts neuronal injury in ischemia-induced stress (Zhou et al., 2017).

One of the putative culprits for neuronal damage during hypoxia may attribute to calcium overload as Ca^2+^ overload results in mitochondrial uncoupling, decreased ATP synthesis, and neuronal death (Chen et al., 2013). Potassium (K^+^) channels are found to be important feedback regulators of the Ca^2+^ influx process. Several types of potassium channels, including apamin-sensitive Ca^2+^-controlled potassium channels activated by the elevated intracellular Ca^2+^ level, further lead to hyperpolarization and abolish neuronal activity and eventually result in neuronal damage (Van Goor et al., 2000). Previously, we have shown that, during chronic HH exposure, SK2 channel expression/activity goes up and hence causes further neurodegeneration (Kushwah et al., 2018). Hence, we further investigated whether GBE-mediated neuroprotection works through SK channel inhibition, and it was found that SK channel expression/activity significantly decreases on GBE treatment which might signify maintenance of calcium homeostasis. Calcium overload is known to cause glutamate excitotoxicity and further lead to apoptosis (Belov et al., 2020), and our observation further validated that GBE treatment prevents glutamate excitotoxicity and apoptosis. Prevention is reproducible and comparable to the group with apamin-sensitive SK2 inhibition that validates the role of SK2 channels in GBE-mediated neuroprotection.

We further explored the putative signaling pathway involved in GBE-mediated neuroprotection. BDNF is well known to promote cell survival and neuroprotection in control conditions as well as in different pathological conditions (Sheng et al., 2018). A study by Kramár et al. showed that BDNF decreases the SK2 component by phosphorylation, causes its inactivation, and strengthens synaptic transmission (Kramar et al., 2004). Therefore, we postulated that GBE-mediated SK2 inhibition may work through the BDNF-mediated signaling mechanism, and it was observed that GBE treatment increases the BDNF level during HH exposure in comparison with groups exposed to HH alone. BDNF overexpression prevents Ca^2+^ overload and shows neuroprotective effects on the neuroglia network under the OGD (oxygen and glucose deprivation) condition (Gaidin et al., 2020). Also, influx of Ca^2+^ regulated different physiological processes through a wide range of target proteins such as ERK, CaMKs, and CREB (Agell et al., 2002), which are vital for neuronal survival. Hence, in the present study, we demonstrated that BDNF further inhibits CaMKII phosphorylation and activates CREB through the ERK pathway. Coherent to several other reports that showed the role of the ERK/CREB pathway in neuroprotection (Park et al., 2004; Xu et al., 2007; Zhao et al., 2018; Zhong et al., 2019), GBE-mediated neuroprotection also involved activation of both the ERK and CREB pathways (Figure 8).

## Conclusion

GBE prevents chronic HH-induced memory impairment and oxidative stress. It further averts neurodegeneration by reducing apoptosis. The neuroprotective effect of GBE may contribute to its efficacy in facilitating BDNF overexpression that further inhibits SK2 channels and reduces cell death. BDNF-mediated SK2 inhibition further activates survival machinery of the cell by activation of the CaMKII/ERK/CREB signaling pathway.

## Data Availability

The original contributions presented in the study are included in the article/Supplementary Material, and further inquiries can be directed to the corresponding author.

## Abbreviations

BDNF, brain-derived neurotrophic factor; BSA, bovine serum albumin; DAB, 3,3′-diaminobenzidine; DCFHDA, 2′,7′-dichlorofluorescein-diacetate; DPX, distyrene, plasticizer, and xylene; GBE, *Ginkgo biloba* L. leaf extract; GSH, glutathione; HA, high altitude; HH, hypobaric hypoxia; MDA, malondialdehyde; PFA, paraformaldehyde; SK channels, small conductance calcium-activated potassium channels; ROS, reactive oxygen species

## References

[B1] Abdel-Wahab B. A. Abd El-Aziz S. M. (2012). Ginkgo Biloba Protects against Intermittent Hypoxia-Induced Memory Deficits and Hippocampal DNA Damage in Rats. Phytomedicine 19 (5), 444–450. 10.1016/j.phymed.2011.11.011 22265820

[B2] Achete de Souza G. de Marqui S. V. Matias J. N. Guiguer E. L. Barbalho S. M. (2020). Effects of Ginkgo Biloba on Diseases Related to Oxidative Stress. Planta Med. 86 (6), 376–386. 10.1055/a-1109-3405 32097975

[B3] Adelman J. P. Maylie J. Sah P. (2012). Small-Conductance Ca2+-Activated K+Channels: Form and Function. Annu. Rev. Physiol. 74, 245–269. 10.1146/annurev-physiol-020911-153336 21942705

[B4] Agell N. Bachs O. Rocamora N. Villalonga P. (2002). Modulation of the Ras/Raf/MEK/ERK Pathway by Ca2+, and Calmodulin. Cell Signal. 14 (8), 649–654. 10.1016/s0898-6568(02)00007-4 12020764

[B5] Anjum V. Arora P. Ansari S. H. Najmi A. K. Ahmad S. (2017). Antithrombocytopenic and Immunomodulatory Potential of Metabolically Characterized Aqueous Extract of Carica Papaya Leaves. Pharm. Biol. 55, 2043–2056. 10.1080/13880209.2017.1346690 28836477 PMC6130488

[B6] Aydin D. Peker E. G. Karakurt M. D. Gurel A. Ayyildiz M. Cevher S. C. (2016). Effects of Ginkgo Biloba Extract on Brain Oxidative Condition after Cisplatin Exposure. Clin. Invest. Med. 39 (6), 27511. 10.25011/cim.v39i6.27511 27917801

[B7] Basnyat B. Murdoch D. R. (2003). High-altitude Illness. The Lancet 361 (9373), 1967–1974. 10.1016/S0140-6736(03)13591-X 12801752

[B8] BelovKirdajova D. Kriska J. Tureckova J. Anderova M. (2020). Ischemia-Triggered Glutamate Excitotoxicity from the Perspective of Glial Cells. Front Cel Neurosci 14, 51. 10.3389/fncel.2020.00051 PMC7098326 32265656

[B9] Belviranl M. Okudan N. (2015). The Effects of Ginkgo Biloba Extract on Cognitive Functions in Aged Female Rats: the Role of Oxidative Stress and Brain-Derived Neurotrophic Factor. Behav. Brain Res. 1, 453–461. 10.1016/j.bbr.2014.10.032 25446810

[B10] Blecharz-Klin K. Piechal A. Joniec I. Pyrzanowska J. Widy-Tyszkiewicz E. (2009). Pharmacological and Biochemical Effects of Ginkgo Biloba Extract on Learning, Memory Consolidation and Motor Activity in Old Rats. Acta Neurobiol. Exp. (Wars) 69, 217–231. 19593336 10.55782/ane-2009-1747

[B11] Chandrasekaran K. Mehrabian Z. Spinnewyn B. Chinopoulos C. Drieu K. Fiskum G. (2002). Bilobalide, a Component of the Ginkgo Biloba Extract (EGb 761), Protects against Neuronal Death in Global Brain Ischemia and in Glutamate-Induced Excitotoxicity. Cel Mol Biol (Noisy-le-grand) 48, 663–669. 12396077

[B12] Chen J. Liao W. Gao W. Huang J. Gao Y. (2013). Intermittent Hypoxia Protects Cerebral Mitochondrial Function from Calcium Overload. Acta Neurol. Belg. 113 (4), 507–513. 10.1007/s13760-013-0220-8 24122478

[B13] Dröge W. (2002). Free Radicals in the Physiological Control of Cell Function. Physiol. Rev. 82, 47–95. 10.1152/physrev.00018.2001 11773609

[B14] Gaidin S. G. Turovskaya M. V. Gavrish M. S. Babaev A. A. Mal’tseva V. N. Blinova E. V. (2020). The Selective BDNF Overexpression in Neurons Protects Neuroglial Networks against OGD and Glutamate-Induced Excitotoxicity. Int. J. Neurosci. 130 (4), 363–383. 10.1080/00207454.2019.1691205 31694441

[B15] Goor F. V. Krsmanovic L. Z. Catt K. J. Stojilkovic S. S. (2000). Autocrine Regulation of Calcium Influx and Gonadotropin-Releasing Hormone Secretion in Hypothalamic Neurons. Biochem. Cel Biol. 78 (3), 359–370. 10.1139/o00-058 10949086

[B16] Hammond R. S. Bond C. T. Strassmaier T. Thu Ngo-Anh T. J. Adelman J. P. Maylie J. (2006). Small-Conductance Ca2+-Activated K+ Channel Type 2 (SK2) Modulates Hippocampal Learning, Memory, and Synaptic Plasticity. J. Neurosci. 26, 1844–1853. 10.1523/JNEUROSCI.4106-05.2006 16467533 PMC6793641

[B17] Hota S. K. Barhwal K. Ray K. Singh S. B. Ilavazhagan G. (2008). Ceftriaxone Rescues Hippocampal Neurons from Excitotoxicity and Enhances Memory Retrieval in Chronic Hypobaric Hypoxia. Neurobiol. Learn. Mem. 89, 522–532. 10.1111/j.1460-9568.2007.0590510.1016/j.nlm.2008.01.003 18304843

[B18] Hota S. K. Barhwal K. Singh S. B. Sairam M. Ilavazhagan G. (2008). NR1 and GluR2 Expression Mediates Excitotoxicity in Chronic Hypobaric Hypoxia. J. Neurosci. Res. 86 (5), 1142–1152. 10.1002/jnr.21554 17969105

[B19] Jain V. Baitharu I. Barhwal K. Prasad D. Singh S. B. Ilavazhagan G. (2012). Enriched Environment Prevents Hypobaric Hypoxia Induced Neurodegeneration and Is Independent of Antioxidant Signaling. Cell Mol Neurobiol 32, 599–611. 10.1007/s10571-012-9807-5 22331403 PMC11498567

[B20] Jain V. Baitharu I. Prasad D. Ilavazhagan G. (2013). Enriched Environment Prevents Hypobaric Hypoxia Induced Memory Impairment and Neurodegeneration: Role of BDNF/PI3K/GSK3β Pathway Coupled with CREB Activation. PLoS One 8, e62235. 10.1371/journal.pone.0062235 23704876 PMC3660501

[B21] Jayalakshmi K. Singh S. B. Kalpana B. Sairam M. Muthuraju S. Ilavazhagan G. (2007). N-acetyl Cysteine Supplementation Prevents Impairment of Spatial Working Memory Functions in Rats Following Exposure to Hypobaric Hypoxia. Physiol. Behav. 92, 643–650. 10.1016/j.physbeh.2007.05.051 17602713

[B22] Kramár E. A. Lin B. Lin C. Y. Arai A. C. Gall C. M. Lynch G. (2004). A Novel Mechanism for the Facilitation of Theta-Induced Long-Term Potentiation by Brain-Derived Neurotrophic Factor. J. Neurosci. 24 (22), 5151–5161. 10.1523/jneurosci.0800-04.2004 15175384 PMC6729196

[B23] Kuiper E. F. E. Nelemans A. Luiten P. Nijholt I. Dolga A. Eisel U. (2012). KCa2 and KCa3 Channels in Learning and Memory Processes, and Neurodegeneration. Front. Pharmacol. 3, 1–13. 10.3389/fphar.2012.00107 22701424 PMC3372087

[B24] Kumari P. Wadhwa M. Chauhan G. Alam S. Roy K. Kumar Jha P. (2020). Hypobaric Hypoxia Induced Fear and Extinction Memory Impairment and Effect of Ginkgo Biloba in its Amelioration: Behavioral, Neurochemical and Molecular Correlates. Behav. Brain Res. 387, 112595. 10.1016/j.bbr.2020.112595 32194184

[B25] Kushwah N. Jain V. Deep S. Prasad D. Singh S. B. Khan N. (2016). Neuroprotective Role of Intermittent Hypobaric Hypoxia in Unpredictable Chronic Mild Stress Induced Depression in Rats. PLoS One 11 (2), e0149309. 10.1371/journal.pone.0149309 26901349 PMC4763568

[B26] Kushwah N. Jain V. Dheer A. Kumar R. Prasad D. Khan N. (2018). Hypobaric Hypoxia-Induced Learning and Memory Impairment: Elucidating the Role of Small Conductance Ca2+-Activated K+ Channels. Neuroscience 388, 418–429. 10.1016/j.neuroscience.2018.07.026 30048783

[B27] LeBel C. P. Ali S. F. McKee M. Bondy S. C. (1990). Organometal-induced Increases in Oxygen Reactive Species: The Potential of 2′,7′-dichlorofluorescin Diacetate as an index of Neurotoxic Damage. Toxicol. Appl. Pharmacol. 104, 17–24. 10.1016/0041-008X(90)90278-3 2163122

[B28] Li Z. Nakaya Y. Niwa Y. Chen X. (2001). KCa Channel-Opening Activity of Ginkgo Biloba Extracts and Ginsenosides in Cultured Endothelial Cells. Clin. Exp. Pharmacol. Physiol. 28, 441–445. 10.1046/j.1440-1681.2001.03456.x10.1046/j.1440-1681.2001.3456.x 11380519

[B29] Liang T. Miyakawa T. Yang J. Ishikawa T. Tanokura M. (2018). Quantification of Terpene Trilactones in Ginkgo Biloba with a 1H NMR Method. J. Nat. Med. 72, 793–797. 10.1007/s11418-018-1203-0 29569220

[B30] Lin M. T. Luján R. Watanabe M. Adelman J. P. Maylie J. (2008). SK2 Channel Plasticity Contributes to LTP at Schaffer collateral-CA1 Synapses. Nat. Neurosci. 11, 170–177. 10.1038/nn2041 18204442 PMC2613806

[B31] Liu P. Zou D. Chen K. Zhou Q. Gao Y. Huang Y. (2016). Dihydromyricetin Improves Hypobaric Hypoxia-Induced Memory Impairment via Modulation of SIRT3 Signaling. Mol. Neurobiol. 53, 7200–7212. 10.1007/s12035-015-9627-y 26687185

[B32] Lugasi A. Dworschák E. Horvatovich P. (1999). Additional Information to theIn Vitro Antioxidant Activity ofGinkgo Biloba L. Phytother. Res. 13 (2), 160–162. 10.1002/(sici)1099-1573(199903)13:2<160::aid-ptr402>3.0.co;2-h 10190193

[B33] Maiti P. Singh S. B. Mallick B. Muthuraju S. Ilavazhagan G. (2008). High Altitude Memory Impairment Is Due to Neuronal Apoptosis in hippocampus, Cortex and Striatum. J. Chem. Neuroanat. 36 (3-4), 227–238. 10.1016/j.jchemneu.2008.07.003 18692566

[B34] Maiti P. Singh S. B. Sharma A. K. Muthuraju S. Banerjee P. K. Ilavazhagan G. (2006). Hypobaric Hypoxia Induces Oxidative Stress in Rat Brain. Neurochem. Int. 49, 709–716. 10.1016/j.neuint.2006.06.002 16911847

[B35] Martin L. Latypova X. Terro F. (2011). Post-translational Modifications of Tau Protein: Implications for Alzheimer's Disease. Neurochem. Int. 58, 458–471. 10.1016/j.neuint.2010.12.023 21215781

[B36] Mazumder A. G. Sharma P. Patial V. Singh D. (2017). Ginkgo Biloba L. Attenuates Spontaneous Recurrent Seizures and Associated Neurological Conditions in Lithium-Pilocarpine Rat Model of Temporal Lobe Epilepsy through Inhibition of Mammalian Target of Rapamycin Pathway Hyperactivation. J. Ethnopharmacology 204, 8–17. 10.1016/j.jep.2017.03.060 28390940

[B37] Mukherjee P. K. Rai S. Bhattacharya S. Wahile A. Saha B. P. (2008). Marker Analysis of Polyherbal Formulation, Triphala - A Well-Known Indian Traditional Medicine. Indian J. Tradit. Knowl. 7, 379–383.

[B38] Muthuraju S. Pati S. (2014). Effect of Hypobaric Hypoxia on Cognitive Functions and Potential Therapeutic Agents. Malays J. Med. Sci. 21, 41–45. 25941462 PMC4405810

[B39] Naik S. R. Pilgaonkar V. W. Panda V. S. (2006). Evaluation of Antioxidant Activity ofGinkgo Biloba Phytosomes in Rat Brain. Phytother. Res. 20 (11), 1013–1016. 10.1002/ptr.1976 16909446

[B40] Park E. M. Joh T. H. Volpe B. T. Chu C. K. Song G. Cho S. (2004). A Neuroprotective Role of Extracellular Signal-Regulated Kinase in N-Acetyl-O-Methyldopamine-Treated Hippocampal Neurons after Exposure to *In Vitro* and *In Vivo* Ischemia. Neuroscience 123 (1), 147–154. 10.1016/j.neuroscience.2003.08.023 14667449

[B41] Sheng S. Huang J. Ren Y. Zhi F. Tian X. Wen G. (2018). Neuroprotection against Hypoxic/Ischemic Injury: δ-Opioid Receptors and BDNF-TrkB Pathway. Cell Physiol Biochem 47 (1), 302–315. 10.1159/000489808 29768254

[B42] Shinozuka K. Umegaki K. Kubota Y. Tanaka N. Mizuno H. Yamauchi J. (2002). Feeding of Ginkgo Biloba Extract (GBE) Enhances Gene Expression of Hepatic Cytochrome P-450 and Attenuates the Hypotensive Effect of Nicardipine in Rats. Life Sci. 70, 2783–2792. 10.1016/S0024-3205(02)01530-8 12269382

[B43] Shukitt-Hale B. Kadar T. Marlowe B. E. Stillman M. J. Galli R. L. Levy A. (1996). Morphological Alterations in the hippocampus Following Hypobaric Hypoxia. Hum. Exp. Toxicol. 15, 312–319. 10.1177/096032719601500407 8845221

[B44] Shukitt-Hale B. Stillman M. J. Welch D. I. Levy A. Devine J. A. Lieberman H. R. (1994). Hypobaric Hypoxia Impairs Spatial Memory in an Elevation-dependent Fashion. Behav. Neural Biol. 62 (3), 244–252. 10.1016/S0163-1047(05)80023-8 7857247

[B45] Vaghef L. Bafandeh Gharamaleki H. (2017). Effects of Physical Activity and Ginkgo Biloba on Cognitive Function and Oxidative Stress Modulation in Ischemic Rats. Int. J. Angiol 26, 158–164. 10.1055/s-0036-1588024 28804233 PMC5552890

[B46] Vick K. A. Guidi M. Stackman R. W. Jr (2010). *In Vivo* pharmacological Manipulation of Small Conductance Ca2+-Activated K+ Channels Influences Motor Behavior, Object Memory and Fear Conditioninguences Motor Behavior, Object Memory and Fear Conditioning. Neuropharmacology 58, 650–659. 10.1016/j.neuropharm.2009.11.008 19944112 PMC3311509

[B47] Wang C. Wang B. (2016). Ginkgo Biloba Extract Attenuates Oxidative Stress and Apoptosis in Mouse Cochlear Neural Stem Cells. Phytother. Res. 30, 774–780. 10.1002/ptr.5572 26799058

[B48] Xu Y. Cui C. Pang C. Christen Y. Luo Y. (2007). Restoration of Impaired Phosphorylation of Cyclic AMP Response Element-Binding Protein (CREB) by EGb 761 and its Constituents in Aβ-Expressing Neuroblastoma Cells. Eu J. Neurosci. 26, 2931–2939. 10.1111/j.1460-9568.2007.05905.x 18001288

[B49] Zhao M. Cheng X. Lin X. Han Y. Zhou Y. Zhao T. (2019). Metformin Administration Prevents Memory Impairment Induced by Hypobaric Hypoxia in Rats. Behav. Brain Res. 363, 30–37. 10.1016/j.bbr.2019.01.048 30703397

[B50] Zhao M. Dong Z. H. Yu Z. H. Xiao S. Y. Li Y. M. (2012). Effects of Ginkgo Biloba Extract in Improving Episodic Memory of Patients with Mild Cognitive Impairment: a Randomized Controlled Trial. J. Chin. Integr. Med.. 10, 628–634. 10.3736/jcim20120605 22704410

[B51] Zhao P. Yang J.-M. Wang Y.-S. Hao Y.-J. Li Y.-X. Li N. (2018). Neuroprotection of Cytisine against Cerebral Ischemia-Reperfusion Injury in Mice by Regulating NR2B-ERK/CREB Signal Pathway. Neurochem. Res. 43 (8), 1575–1586. 10.1007/s11064-018-2572-1 29948728

[B52] Zhong X. Li G. Qiu F. Huang Z. (2019). Paeoniflorin Ameliorates Chronic Stress-Induced Depression-like Behaviors and Neuronal Damages in Rats via Activation of the ERK-CREB Pathway. Front. Psychiatry 9, 772. 10.3389/fpsyt.2018.00772 30692946 PMC6339947

[B53] Zhou X. Wang H.-Y. Wu B. Cheng C.-Y. Xiao W. Wang Z.-Z. (2017). Ginkgolide K Attenuates Neuronal Injury after Ischemic Stroke by Inhibiting Mitochondrial Fission and GSK-3β-dependent Increases in Mitochondrial Membrane Permeability. Oncotarget 8, 44682–44693. 10.18632/oncotarget.17967 28591721 PMC5546510

[B54] Zhu B. T. Taneja N. Loder D. P. Balentine D. A. Conney A. H. (1998). Effects of tea Polyphenols and Flavonoids on Liver Microsomal Glucuronidation of Estradiol and Estrone. J. Steroid Biochem. Mol. Biol. 64, 207–215. 10.1016/S0960-0760(97)00163-5 9605416

