# Supplementary figures and images for: Ginkgo biloba L. Prevents Hypobaric Hypoxia–Induced Spatial Memory Deficit Through Small Conductance Calcium-Activated Potassium Channel Inhibition: The Role of ERK/CaMKII/CREB Signaling
Source: Front Pharmacol. 2021 Jul 12;12:669701. doi: 10.3389/fphar.2021.669701 (PMC8313424; doi:10.3389/fphar.2021.669701)

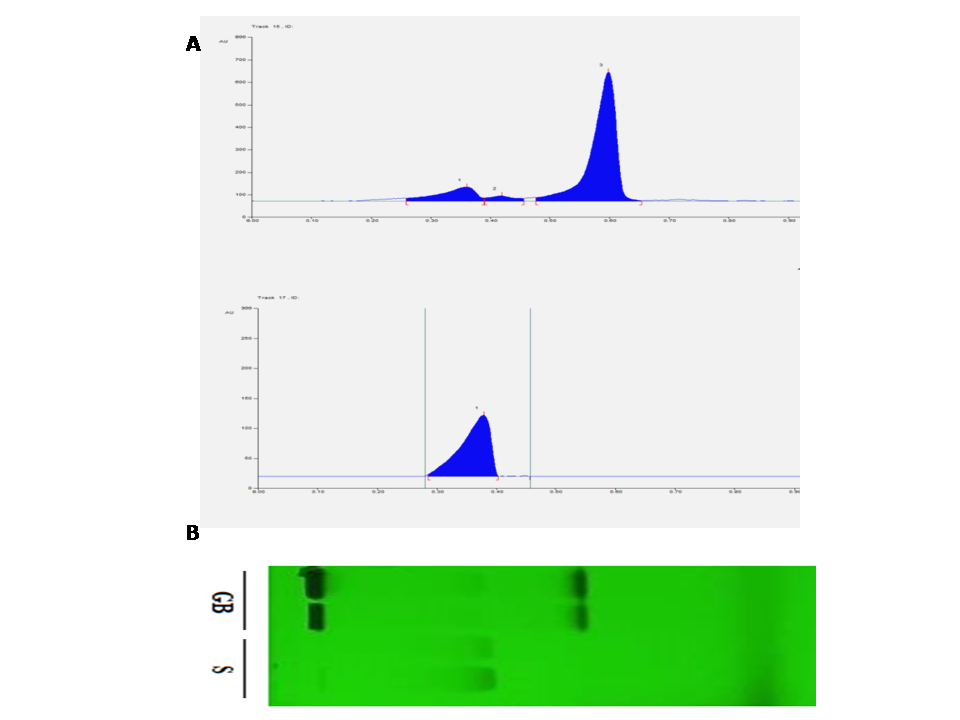

Supplement: Supplementary file 1 [file Image1.TIF]
